# Proteomic Characterization of Differential Abundant Proteins Accumulated between Lower and Upper Epidermises of Fleshy Scales in Onion (*Allium cepa* L.) Bulbs

**DOI:** 10.1371/journal.pone.0168959

**Published:** 2016-12-30

**Authors:** Si Wu, Fen Ning, Xiaolin Wu, Wei Wang

**Affiliations:** College of Sciences, Henan Agricultural University, Zhengzhou, China; Youngstown State University, UNITED STATES

## Abstract

The onion (*Allium cepa* L.) is widely planted worldwide as a valuable vegetable crop. The scales of an onion bulb are a modified type of leaf. The one-layer-cell epidermis of onion scales is commonly used as a model experimental material in botany and molecular biology. The lower epidermis (LE) and upper epidermis (UE) of onion scales display obvious differences in microscopic structure, cell differentiation and pigment synthesis; however, associated proteomic differences are unclear. LE and UE can be easily sampled as single-layer-cell tissues for comparative proteomic analysis. In this study, a proteomic approach based on 2-DE and mass spectrometry (MS) was applied to compare LE and UE of fleshy scales from yellow and red onions. We identified 47 differential abundant protein spots (representing 31 unique proteins) between LE and UE in red and yellow onions. These proteins are mainly involved in pigment synthesis, stress response, and cell division. Particularly, the differentially accumulated chalcone-flavanone isomerase and flavone O-methyltransferase 1-like in LE may result in the differences in the onion scale color between red and yellow onions. Moreover, stress-related proteins abundantly accumulated in both LE and UE. In addition, the differential accumulation of UDP-arabinopyranose mutase 1-like protein and β-1,3-glucanase in the LE may be related to the different cell sizes between LE and UE of the two types of onion. The data derived from this study provides new insight into the differences in differentiation and developmental processes between onion epidermises. This study may also make a contribution to onion breeding, such as improving resistances and changing colors.

## Introduction

The onion (*Allium cepa* L.) is a member of the *Liliaceae* family and is widely planted worldwide as a valuable vegetable crop, with fleshy scales that form bulbs as the main edible parts. The onion bulb has a unique flavor and a long storage period. It contains important dietary flavonoids and alk(en)yl cysteine sulfoxides and has many activities that are beneficial to human health, e.g., anticancer properties, and antiplatelet, antithrombotic and anti-asthmatic activities [1]. Global onion production increased from 52.65 million tons in 2003 to 85.79 million tons in 2013; In 2013, China’s onion production reached 22.34 million tons and ranked first place worldwide (FAO). Currently, onions are the second most-grown vegetable in the world after tomatoes.

The scales of an onion bulb are modified leaves. The edges of the scales bond together around the bud and finally form the bulb. Due to the polarity of the leaf primordia during differentiation and development, onion scales also show dorsoventrality. Each scale consists of an upper epidermis (UE), an intermediate parenchyma tissue (mesophyll), and a lower epidermis (LE). The epidermis of onion scales forms a boundary between the plant and the external environment. It serves different functions, such as protecting against water loss, regulating gas exchange, secreting metabolic compounds, and absorbing water and mineral nutrients.

Previous studies showed that cell differentiation capacity and maintenance of differentiated phenotypes (e.g., stomata and trichome cells) in plant epidermis were regulated through the coordination of multiple genes (e.g., defective kernel 1, homeodomain-leucine zipper, *Arabidopsis thaliana* meristem 1, protodermal factor 2) [2–5]. However, the differentiation mechanisim between LE and UE in onion has largely unclear.

Both LE and UE in onion are simple tissues, consisting of one layer of closely-associated cells that play a mechanical, protective role. UE is colorless, transparent and easily peels off, whereas LE is yellow, red or white in color, due to flavonoid compounds (e.g., quercetin, anthocyanin) [6, 7]. Onion bulbs are categorized as yellow, red (purple), and white based on the color of their scales. In yellow and red onions, quercetin is the most abundant flavonoid [7]. Anthocyanins represent approximately 10% of the total flavonoids, with cyanine derivatives as the major anthocyanins among at least 25 different compounds [6, 8].

LE and UE display obvious differences in cell differentiation and pigment synthesis. Unique microscopic structures and physiological functions make the epidermis of onion scales a commonly used experimental material in botany and molecular biology. The epidermis of onion scales has been used in biosensors [9], transient transformation (detecting protein subcellular localization) [10], cellular and subcellular metabolite analysis [11], and growth anisotropy [12]. However, the associated proteomic differences between LE and UE are unclear. The onion epidermis is a suitable material to carry out single-layer-cell proteomic analysis.

Although onion genome sequencing has not been reported and the number of identified proteins in the onion is limited in databases, the increasing numbers of available expressed sequence tag (EST) databases and the high homology of the onion to the lily genome allow the successful identification of proteins, as shown by a recent research study [13]. The present study compared the proteomic differences between LE and UE in yellow and red onion scales using 2-DE followed by matrix-assisted laser desorption ionization-time of flight (MALDI-TOF) Mass Spectrometry (MS), revealing a number of the differential accumulated proteins involved in pigment synthesis, stress response and cell division in the onion epidermis. Our results provided new insight into the differences in differentiation and developmental processes between onion epidermises. This study may also make a contribution to onion breeding, such as improving resistances and changing colors.

## Materials and Methods

### Plant materials

**Fig 1:**
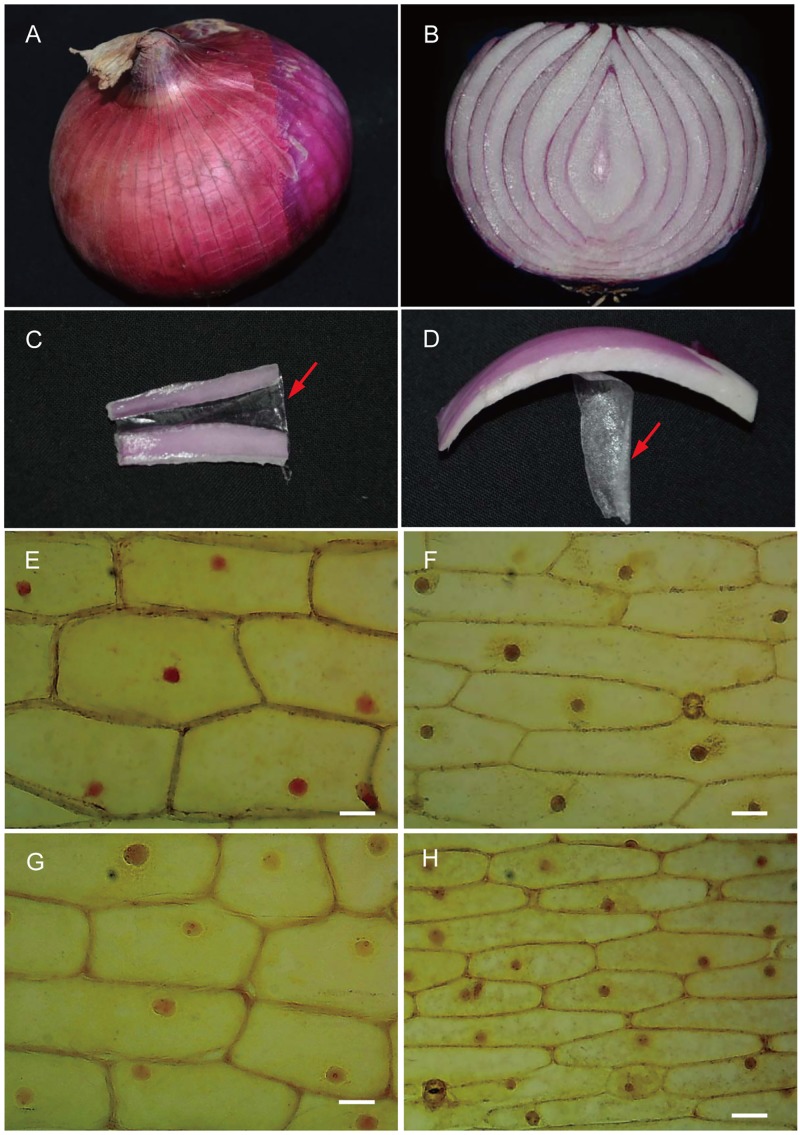
The epidermises of onion scales. (A) Red onion bulb. B, Longitudinal section of a red onion scale. (C) The lower epidermis (LE) (*arrow*). (D) The upper epidermis (UE) (*arrow*). (E-H) Light microscopy of the LE (F and H) and UE (E and G) from a yellow onion scale (E and F) and a red onion scale (G and H). The bar = 2.0 μm.

Mature bulbs of yellow and red onions with diameter approximately 7–9 cm were obtained from a local market (Henan, Zhengzhou, China). The fleshy scales of onion bulbs were cut into pieces, and LE and UE were manually separated (Fig 1). For one independent biological experiment, the samples were collected from one onion, in which the outer dry leaves and inner light-colored leaves (about 3–4 layers) were discarded. Fresh samples were immediately used for microscopic observation or frozen in liquid nitrogen and stored at -80°C until the pigment assay and proteomic analysis.

### Microscopic observation

Onion scale epidermises were stained with I_2_-KI and mounted onto temporary slides in Milli-Q water for light microscopy observation. The stained specimens were observed using a Phoenix PH50 microscope fitted with 4X lenses and were recorded and digitized using ToupView x86 software.

### Pigment assay

Onion scale epidermises were ground to fine powder in liquid nitrogen, and homogenized in a mortar in distilled water (1 g/10 ml). The crude extract was shaken for 3–4 h [14] and then clarified through centrifugation. The absorbance spectra of pigment extracts were analyzed using a spectrophotometer UV-2550 (Shimadzu, Japan). The absorbance spectra were further recorded after titrating with 1 N HCl or 1 N NaOH.

### Protein extraction and quantitation

Onion scale epidermises (ca. 1.0 g) were ground to fine powder in liquid nitrogen. Protein was extracted using the extraction solution (0.1 M Tris-HCl, pH 8.8, 5 mM DTT and 2 mM PMSF, 1.0 g/5 ml), and then subjected to phenol extraction as described previously [15]. For protein assay, protein precipitates were dissolved in a 2DE rehydration buffer without the IPG buffer to avoid its interference [16]. Protein concentrations were determined using Bradford method with bovine serum albumin as a standard.

### 2-DE and MS/MS

Approximately 800 μg of proteins were loaded into 11 cm linear pH 4–7 IPG strips in the Ettan III system (GE Healthcare, USA). 2-DE was performed as described before [17]. Digital images of the gels were analyzed using PDQuest 8.0 software (Bio-Rad, USA). The gels from three independent replicates were analyzed. Protein spots with at least 2-fold abundance change between the epidermises were excised from the gels, then reduced (10 mM DTT), alkylated (50 mM iodoacetic acid) and subsequently digested with 10 mg/ml of trypsin for 16 h at 37°C in 50 mM ammonium bicarbonate. The supernatants were vacuum-dried and dissolved in 5 μl 0.1% TFA followed by mixing in 1:1 ratio with a matrix consisting of a saturated solution of α-cyano-4-hydroxy-trans-cinnamic acid in 50% CAN and 1% TFA. The digested fragments were analyzed using a MALDI-TOF/TOF analyzer (Applied Biosystems, Foster City, USA). The MS/MS spectra were acquired in the positive ion mode and automatically submitted to Mascot 2.3 search engine (Matrix Science Ltd., London, U.K.) for peptide mass finger printing against the NCBInr 20150320 database (species, onion; protein database, 21872 sequences; EST database, 121224 sequences); type of search, MALDI-TOF ion search; enzyme, trypsin; variable modifications, acetyl (protein N-term), deamidated (NQ), dioxidation (W) and oxidation (M); fixed modifications, carbamidomethyl (C); mass values monoisotopic; protein mass, unrestricted; peptide mass tolerance, 100 ppm; fragment mass tolerance, 0.5 Da; max missed cleavages, 1; and instrument type, MALDI-TOF-TOF. Only significant scores greater than ‘identity’ defined through the probability analysis were considered for assigning protein identity. All of the positive protein identification scores were significant (p<0.05, score>32).

### Bioinformatic analysis

By searching the key word “*Allium cepa*”, 1049 protein entries and 20204 ESTs entries were retrieved in the NCBInr database (version 20160428), and 364 onion protein entries retrieved in the UniProtKB database (version 20160428). This sequence information, together with homologous protein sequences from related monocotyledons (e.g., lily), allow for the successful identification of onion proteins by MS/MS [13]. Moreover, a BLASTX search can be applied to search for homologous proteins corresponding to onion ESTs from monocotyledons, such as liliaceous plants, maize, or rice.

### Enzyme activity assay

The fresh onion epidermises were ground with 15 mM sodium acetate (pH 5.0, 0.2 g/1.0 ml). The homogenate was centrifuged at 12,000 g for 10 min. The supernatant was used for β-1,3-glucanase activity assay [18]. One unit of β-1,3-glucanase activity was defined as the concentration of the enzyme that produced 1 μmol glucose equivalents.

### Semi-quantitative RT-PCR

The mRNA sequences of the onion chalcone-flavanone isomerase (*CHI*, AY541034.1) and *actin* (GU570135.2) were retrieved in the NCBI database. RNA was extracted from the epidermises of onion scales using TRIzol reagent (CW Biotech, China). cDNA was made with a HiFi-script first strand cDNA synthesis kit (CW Biotech, China). Primer sequences were *actin*F, AGAGCAGTATTCCCAAGC; *actin*R, TCTTCAGGAGCAACACGA; *CHI*F, TTTACGGCAATCGGCATCTA; *CHI*R, GCTTAGC AGCAGGTGATACA.

### Statistical analysis

The results of the microscopic observation and enzyme assay were the means of three replicates. The mean values were compared by one-way analysis of variance with post hoc multiple comparisons using Fisher's least significant difference test (p < 0.05).

## Results and Discussion

### Comparative proteomic analysis of onion epidermises

**Fig 2:**
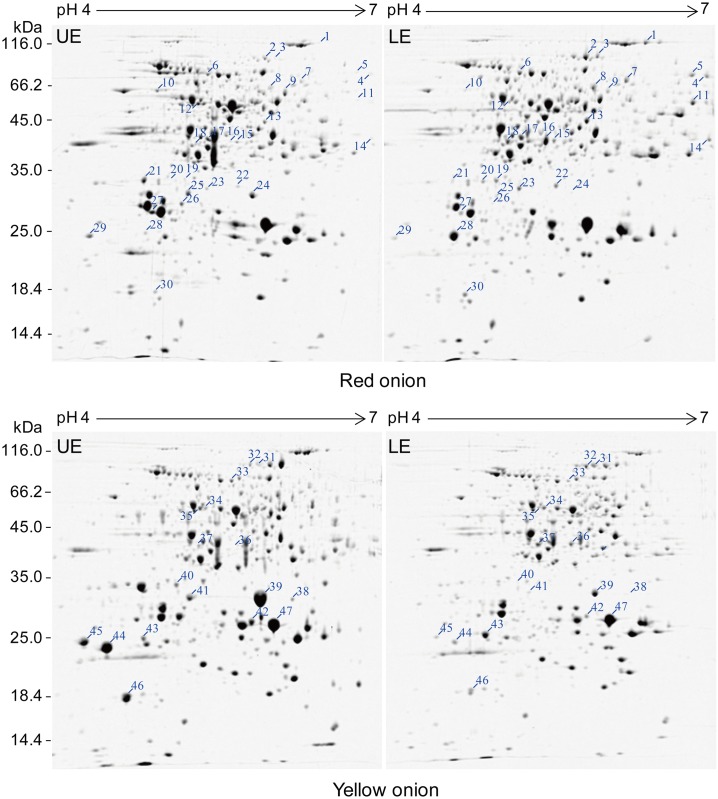
2-DE detection of differentially abundant proteins between LE and UE in onion scales. CBB-stained gels from two independent experiments. Differential spots with at least two-fold changes in volume are indicated.

The protein yield in UE was higher than that in LE, and in red onion scale was higher than that in yellow one (Table 1). In 2-DE experiments, approximately 700–850 CBB-stained protein spots were detected in a range of pH 4–7 and 15–95 kDa, for both onions (Fig 2, Table 1, S1 Fig).

**Table 1:** Protein yield and 2-DE resolved spot number of epidermises in red and yellow onion scales.

Onion types	Protein yield (mg/g dry weight)		Spot number (match rate)^a^	
	UE	LE	UE	LE
Red	5.91 ± 0.11	5.02 ± 0.19	764 ± 9 (66 ± 6%)	845 ± 12 (98 ± 2%)
Yellow	5.62 ± 0.06	4.78 ± 0.06	703 ± 18 (45 ± 4%)	742 ± 10 (51 ± 5%)

^a^ Spot number and match rate were detected using automatic spot detection module of PDQuest software (Bio-Rad), using the gel image of LE of red onion scale as the master gel.

A total of 62 CBB-stained protein spots in 2DE gels were detected with at least 2-fold abundance change between LE and UE in two types of onions from three biological replicates and selected for MS/MS identification. As a result, 47 protein spots were successfully identified (S1 File) and classified into 31 unique proteins, including 30 spots from red onion and 17 spots from yellow onion (Table 2). Among them, 4 spots (spots 9, 27, 28 and 43) were matched with distinct onion proteins, and 43 spots (27 in red onion and 16 in yellow onion) were matched with onion ESTs. The identified proteins were further characterized according to their homologs (in monocots, especially the lily family) in UniProtKB. All 47 protein spots were matched with their homologs at > 80% similarities. The identified proteins were classified into six categories according to the biological process involved (Table 3). Regarding subcellular location, 40% of the identified proteins existed in the cytoplasm, approximately 30% in the vacuole and extracellular space, 20% in plastids, and 10% in other compartments.

**Table 2:** The MS/MS identification of differentially abundance proteins between UE and LE in onion scales.

Spot	Ratio ^a^	NCBI accession	Protein name	Theorial Mr/ p*I*	Mascot Score	Coverage (%)	Matched peptides
**Red scale**							
1	-8.1	CF449626	Translation elongation factor EF-2 subunit	93.92/6.00	395	26	DGNEYLINLIDSPGHVDFSSEVTAALR; ITDGALVVVDCIEGVCVQTETVLR; CFLELQVDGEEAYQTFSR
2	-8.6	CF446696	5-methyltetrahydropteroyltriglutamate-homocysteine methyltransferase	84.67/5.93	296	21	EVEDLEAAGVTVIQIDEAALR; SEHAFYLDWAVHSFR; YGAGIGPGVYDIHSPR
3	-6.4	CF444585	5-methyltetrahydropteroyltriglutamate-homocysteine methyltransferase	84.67/5.93	493	27	KLNLPILPTTTIGSFPQTVELR; EVEDLEAAGVTVIQIDEAALR; GMLTGPVTILNWSFVR; SEHAFYLDWAVHSFR
4	-6.9	CF448000	Malic enzyme	62.93/5.52	183	15	VHDELLLAASEALANEVTQEHYEK; GLIYPPFTNIR
5	-7.6	CF448000	Malic enzyme	62.93/5.52	201	19	VHDELLLAASEALANEVTQEHYEK; GLIYPPFTNIR; VYELGLATR
6	-1.0	CF449636	2,3-bisphosphoglycerate-independent phosphoglycerate mutase	62.29/5.47	178	16	AVGPIVDGDAVVTINFR; ALEYEDFNMFDR; YLVTPPEIER
7	-2.5	CF451841	D-3-phosphoglycerate dehydrogenase	64.40/5.89	123	21	GLGMHVIAHDPYAPADR; GGVIDEDALVR; IFNDETFGK
8	-4.0	CF437616	D-3-phosphoglycerate dehydrogenase	64.80/6.73	253	26	EAQEGVAIEIAEAVVGALK; GLIEPISDTHINLVNADFTAK; VLLDGSPENPLETIR; FASAMSETGDIR
9	1.5	ADA85889	ATPase alpha subunit	55.12/6.11	329	25	VYGLNEIQAGEMVEFASGVK; GIALNLENENVGIVVFGSDTAIK; MTNFYTNFQVDEIGR; DNSMHALIIYDDLSK; EAFPGDVFYLHSR; AVDSLVPIGR; TAIAIDTILNQK; AAELTTLLESR; VVSVGDGIAR
10	1.9	CF437834	Vacuolar ATP synthase subunit B	54.06/5.07	245	26	AVVGEEALSSEDLLYLEFLDKFER; KFVTQGAYDTR; TLDSFYSR
11	-10.2	BQ580030	Serine hydroxymethyltransferase	51.63/6.87	145	21	YYGGNEFIDQIENMCR;VSLETGYIDYDKMEEK; YSEGMPGNR
12	1.8	ES449575	Enolase	48.13/5.59	222	26	GNPTVEADCHLSDGTLAR; AAVPSGASTGVYEALELR
13	-1.4	CF438937	GDP-mannose 3', 5'-epimerase 1	42.78/5.74	221	24	VVGTQAPVQLGSLR; SFTFIDECVEGVLR; KLPIHHIPGPEGVR; EKAPAAFCR; ITYFWIK
14	-3.1	CF443678	Formate dehydrogenase 1	41.45/6.87	220	28	AAAEAGLTVAEVTGSNTVSVAEDELMR; ANEYAAMNPNFVGCVEGSLGIR; NFLPGYHQVVNGEWNVAAIAHR
15	-1.3	CF441553	Glutamine synthetase root isozyme 5	39.26/5.52	100	20	GGNNILVMCDCYTPAGEPIPTNHR; IIAEYIWIGGSGMDIR; AAEIFSHPDVVK
16	-7.1	CF443460	Trans-resveratrol di-O-methyltransferase-like	40.27/5.38	336	32	ELHNAQTHLWNILYNFMNSQTLK; SEQPSTAFEMYHGLYYWDATAK; WVLMPWSDEECVK; VILEDCADQFEGVK; CTLFELPHVIDAIEK
17	-1.0	CF444706	Flavone O-methyltransferase 1-like	39.61/5.62	289	27	AGPNAHLSPSQIADQLPTENPQAPVMIDR; AYGMSAFEYHGTDPR; AAIELDLFEIIKK; ILMESWYYLK
18	-3.4	CF448455	UDP-arabinopyranose mutase 1-like	41.12/5.67	97	15	NLLSPSTPYFFNTLYDPYTEGADFVR; VINVPEGFDYELYNR
19	-4.0	CF437496	Momilactone A synthase-like	27.92/5.73	282	37	FGHLDIMFNNAGITGPAIPDTTSYPLIDFKR; LFIQHGAQIVVADVQDK; VALITGGASGIGER; VIDINVTGPFLGIK
20	-5.5	CF437496	Momilactone A synthase-lik	27.92/5.73	118	13	VALITGGASGIGER; VIDINVTGPFLGIK
21	5.7	CF435027	Chitinase, partial	31.73/5.18	143	12	QEQGNPPDYCQPSTQYPCAPGKK
22	-1.7	CF437643	Chitinase	33.58/4.88	176	18	GFYTYDAFIAAANSFIGFGTTGDTDTR; WSPSAADIGANR; NENSCPAR
23	-2.1	CF437496	Momilactone A synthase-like	27.92/5.73	446	37	FGHLDIMFNNAGITGPAIPDTTSYPLIDFKR; LFIQHGAQIVVADVQDK; VALITGGASGIGER; VIDINVTGPFLGIK
24	10.2	CF440559	Beta-1,3-glucanase	35.40/4.26	87	26	VSTAVSLAVLGPSYPPSMGAFTSDAAQYLQPIVK; NNIQNYPSVSFK; SNSNFNGVR
25	6.3	CF445999	Beta-1,3-glucanase	35.40/4.26	118	24	IVVSESGWPSAGGFAATVGNAQTYNTNLVNHVGK;GQGIESNFGLYYPNK
26	3.5	CF438948	Salicylic acid-binding protein 2-like	29.35/5.46	267	45	RIEEVGTFSEYSQPLLNIMSSLPPNEK; TGLLYIEELSETPAFSK; VTVPDLAASGIDSR; CPEDLAITEDIQEK; LTAILFGPNFFASR; VAHVLESLGHR; EDIMLASTLVR; ENYGSIPR
27	2.3	BAC21275	lachrymatory factor synthase	19.33/5.02	33	11	MELNPGAPAVVADSANGAR
28	-4.6	AAS48418	Chalcone isomerase	24.27/5.10	541	58	ALTQAVLESIIGEHGVSPAAK; LDVEGTAFDSVIIPPGSSK; FTNVTMILPLTGEQYSEK; AIGIYTDAEASAVDKFK; TGEELAGSLDFFR; VTENCVAYWK; THFLGGAGVR
29	2.9	CF450045	Pathogenesis-related protein 5	23.53/4.98	318	27	QLNSGDSWTVTANAGTTGGR; TDEYCCNSGSCGPTDFSR; CTADVNGQCPAQLR; AAGGCNNPCTVFK
30	-1.5	CF438143	Peroxiredoxin-5	17.32/4.85	349	45	VILFGVPGAFTPTCSMQHVPGFISSAEVLK; VANIEQGGEFTISGAEEILK; AKGVDEILLVSVNDPFVMK; YTQELGLELDLTEK; FALLADDLKVK; RFALLADDLK
Yellow scale							
31	-3.9	CF444585	5-methyltetrahydropteroyltriglutamate-homocysteine methyltransferase	84.66/5.93	516	27	LNLPILPTTTIGSFPQTVELR; EVEDLEAAGVTVIQIDEAALR; LQEELDIDVLVHGEPER; SEHAFYLDWAVHSFR
32	-2.3	CF444585	5-methyltetrahydropteroyltriglutamate-homocysteine methyltransferase	84.66/5.93	532	27	KLNLPILPTTTIGSFPQTVELR; EVEDLEAAGVTVIQIDEAALR; LQEELDIDVLVHGEPER; SEHAFYLDWAVHSFR
33	-1.6	CF449636	2,3-bisphosphoglycerate-independent phosphoglycerate mutase	60.29/5.47	239	23	NSDQYLPPFVIVDESGK; AVGPIVDGDAVVTINFR; ALEYEDFNMFDR; YLVTPPEIER
34	-1.9	CF445718	Enolase1	40.08/5.27	256	18	YGQDATNVGDEGGFAPNIQENKEGLELLK; SFVADYPIVSIEDPFDQDDWTTYAK; LAMQEFMILPTGASSFK
35	1.6	ES449575	Enolase	48.13/5.59	179	26	AAVPSGASTGVYEALELR; GNPTVEADCHLSDGTLAR
36	-8.2	CF443460	Trans-resveratrol di-O-methyltransferase-like	40.27/5.38	122	19	SEQPSTAFEMYHGLYYWDATAK; VILEDCADQFEGVK; CTLFELPHVIDAIEK
37	-2.5	BQ580186	Protein disulfide isomerase-like 2–2	39.82/5.67	116	33	SEEDVVIANLDADNYKDLAEK; AGEDYDGGREVDDFVTFINEK; GQLTSQAGVVASLDTIVK; EVDDFVTFINEK; YGVSGYPTLK
38	2.5	CF444510	Hydroxyacylglutathione hydrolasecytoplasmic -like isoform X1	28.59/5.55	64	12	EGEDPAVFTGDTLFVAGCGK; FAATVEPENEK; VFCGHEYTEK; GHISYYVTSK
39	11.5	CF445999	Beta-1,3-glucanase	35.40/4.26	247	26	IVVSESGWPSAGGFAATVGNAQTYNTNLVNHVGK; GQGIESNFGLYYPNKQPVYSI
40	3.9	CF435027	Chitinase	31.73/5.18	98	28	GFYTYDAFIAAANSFSGFGTTGDTNTQKR; QEQGNPPDYCQPSTQYPCAPGKK
41	7.2	CF445999	Beta-1,3-glucanase	35.40/4.26	90	26	IVVSESGWPSAGGFAATVGNAQTYNTNLVNHVGK; GQGIESNFGLYYPNKQPVYSI
42	1.6	CF444732	Thaumatin-like protein 1b	33.37/5.20	218	23	FSCTSGDCGTGQVACNNAGGR; SACEAFNTDEYCCR; SWGTGGYLAGCK; AIGCYVDINAR
43	-2.5	AAS48418	Chalcone isomerase	24.27/5.10	558	51	LDVEGTAFDSVIIPPGSSKTHFLGGAGVR
44	12.8	CF450045	Pathogenesis-related protein 5	23.53/4.98	409	27	QLNSGDSWTVTANAGTTGGR; TDEYCCNSGSCGPTDFSR; CTADVNGQCPAQLR; AAGGCNNPCTVFK
45	3.1	CF450045	Pathogenesis-related protein 5	23.53/4.98	362	21	QLNSGDSWTVTANAGTTGGR; TDEYCCNSGSCGPTDFSR; CTADVNGQCPAQLR
46	3.7	CF443478	Intracellular pathogenesis-related protein	16.90/6.51	139	9	AAPEVIASSTVLSGQGEVGSIR
47	-2.4	CF446666	Tau glutathione S-transferase	25.69/6.24	127	7	FWANYVDSKVWDAGANIWK

^a.^ Log2 ratio of relative abundance (UE/LE), Log2 ratio > 1 represents abundance change more than two folds.

**Table 3:** The differential proteins identified in the epidermises in red onion (spots 1–30) and yellow onion (spots 31–47).

Spot	Homolog proteins(Plant species)	Accession ^a^	Location ^b^	Molecular function
***Proteins in increased abundance in LE***				
*Pigment biosynthesis*				
17	Flavone O-methyltransferase 1-like (*Musa acuminata*)	M0SIB3	*Plastid	O-methyltransferase activity
28	Chalcone-flavanone isomerase (*Allium cepa*)	Q6QHK0	Tonoplast, Nucleus, ER	Secondary metabolite (e.g., flavonoids) biosynthesis
43	Chalcone-flavanone isomerase (*A*. *cepa*)	Q6QHK0	Tonoplast, Nucleus, ER	Secondary metabolite (e.g., flavonoids) biosynthesis
*Stress response*				
13	GDP-mannose 3',5'-epimerase 1 (*Oryza sativa*)	A3C4S4	*Cytoplasm	GDP-mannose 3,5-epimerase activity, coenzyme binding
14	Formate dehydrogenase 1 (*O*. *brachyantha*)	J3ME94	Plastid, Thylakoid	Oxidoreductase activity, NAD binding
16	Trans-resveratrol di-O-methyltransferase-like (*Elaeis guineensis*)	UPI00057A5A6F	*Cytoplasm, Plastid, Mitochondrion	Trans-resveratrol di-O-methyltransferase
19, 20, 23	Momilactone A synthase-like (*Zea mays*)	A0A096R062	*Plastid	Oxidoreductase activity
22	Chitinase (*Allium sativum*)	Q38777	*Extracellular space, Vacuole	Chitinase activity, chitin binding
30	Peroxiredoxin-5 (*Z*. *mays*)	B4FN24	*Cytoplasm	Oxidoreductase activity
36	Trans-resveratrol di-O-methyltransferase-like (*E*. *guineensis*)	UPI00057A5A6F	*Cytoplasm, Plastid, Mitochondrion	Trans-resveratrol di-O-methyltransferase
47	Tau glutathione S-transferase (*A*. *cepa*)	F2ZC01	*Cytoplasm	Transferase activity
*Carbohydrate metabolism*				
4, 5	Malic enzyme (*O*. *sativa*)	Q6T5D1	*Cytoplasm, Plastid	Malate dehydrogenase (NAD^+^) (decarboxylating) activity, metal ion binding
6	2,3-bisphosphoglycerat independent phosphoglycerate mutase (*Z*. *mays*)	C0HHU2	Cytoplasm	Phosphoglycerate mutase activity, Mg^2+^ binding
33	2,3-bisphosphoglycerat independent phosphoglycerate mutase (*Z*. *mays*)	C0HHU2	Cytoplasm	Phosphoglycerate mutase activity, Mg^2+^ binding
34	Enolase1 (*Z*. *mays*)	K7V794	Phosphopyruvate hydratase complex	Phosphopyruvate hydratase activity, Mg^2+^ binding
*Cell division*				
18	UDP-arabinopyranose mutase 1-like (*O*. *brachyantha*)	J3LQM6	Golgi apparatus, Cytoplasm	Intramolecular transferase activity
*Protein synthesis*				
1	Translation elongation factor EF-2 subunit (*Z*. *mays*)	B6U0S1	*Cytoplasm	GTPase activity, translation elongation factor activity, nucleotide binding
2, 3	5-methyltetrahydropteroyltriglutamate-homocysteine methyltransferase 2 (*O*. *sativa*)	Q2QLY4	*Cytosol	5-methyltetrahydropteroyltriglutamate-homocysteine S-methyltransferase activity, Zn^2+^ binding
7	D-3-phosphoglycerate dehydrogenase (*O*. *sativa*)	A3BE72	*Plastid	Amino acid binding, NAD binding, phosphoglycerate dehydrogenase activity
8	D-3-phosphoglycerate dehydrogenase (*Z*. *mays*)	A0A096R8F6	*Plastid	Amino acid binding, NAD binding, phosphoglycerate dehydrogenase activity
11	Serine hydroxymethyltransferase (*O*. *brachyantha*)	J3N865	*Plasmodesma Cytoplasm, Membrane	Pyridoxal phosphate binding, glycine hydroxymethyltransferase activity
15	Glutamine synthetase root isozyme 5 (*Z*. *mays*)	P38563	Cytoplasm	ATP binding, glutamate ammonia ligase activity
31, 32	5-methyltetrahydropteroyltriglutamate-homocysteine methyltransferase 2 (*O*. *sativa*)	Q2QLY4	Cytosol	5-methyltetrahydropteroyltriglutamate-homocysteine S-methyltransferase activity, Zn^2+^ binding
37	Protein disulfide isomerase like 2–2 (*M*. *acuminata*)	M0RS28	ER	Isomerase activity
***Proteins in increased abundance in UE***				
*Stress response*				
21	Chitinase, partial (*A*. *sativum*)	Q38776	*Vacuole, Extracellular space	Chitinase activity, chitin binding
24, 25	Beta-1,3-glucanase (*Lilium*)	Q1W6B9	*Extracellular space	Hydrolase activity, hydrolyzing O-glycosyl compounds
26	Salicylic acid-binding protein 2-like (*E*. *guineensis*)	UPI00057B4D46	*Plastid	Lipase activity, methyl salicylate esterase activity
29	Pathogenesis-related protein 5(*A*. *sativum*)	A0A0K1L9H1	*Vacuole	Pathogenesis-related protein, plant defense
38	Hydroxyacylglutathione hydrolase cytoplasmic-like isoform X1 (*O*. *brachyantha*)	J3LNG8	Cytosol	Hydroxyacylglutathione hydrolase activity
39, 41	Beta-1,3-glucanase (*Lilium*)	Q1W6B9	*Extracellular space	Hydrolase activity, hydrolyzing O-glycosyl compounds
40	Chitinase (*A*. *sativum*)	Q38777	*Vacuole, Extracellular space	Chitinase activity, chitin binding
42	Thaumatin-like protein 1b (*Z*. *mays*)	A0A096QLD7	*Vacuole	Allergenic/antifungal thaumatin-like proteins
44, 45	Pathogenesis-related protein 5 (*A*. *sativum*)	A0A0K1L9H1	Vacuole	Pathogenesis-related protein, plant defense
46	Intracellular pathogenesis related protein (*Asparagus officinalis*)	Q9SB87	*Cytoplasm	Pathogenesis-related protein
*Carbohydrate metabolism*				
9	ATP synthase subunit alpha (*A*. *cepa*)	D2XT90	Mitochondrion	Proton-transporting ATPase activity, ATP binding
10	Vacuolar ATP synthase subunit B (*Z*. *mays*)	B6T9C0	Vacuole	ATP binding, hydrolase activity
12	Enolase (*Z*. *mays*)	B6T3P9	Phosphopyruvate hydratase complex	Phosphopyruvate hydratase activity, Mg^2+^ binding
35	Enolase (*Z*. *mays*)	B6T3P9	Phosphopyruvate hydratase complex	Phosphopyruvate hydratase activity, Mg^2+^ binding
*Others*				
27	Lachrymatory factor synthase (*A*. *cepa*)	P59082	Vacuole	Lachrymatory factor synthase activity

^a^ UniProtKB/UniRef accession;

^b^ Subcellular localization of proteins annotated in UniProtKB/Swiss-Prot (http://www.uniprot.org/) except those with asterisk mark which were predicte d in online Plant-mPLoc server (http://www.csbio.sjtu.edu.cn/bioinf/plant-multi/).

### Differential protein expression related to pigment synthesis in onion epidermises

**Fig 3:**
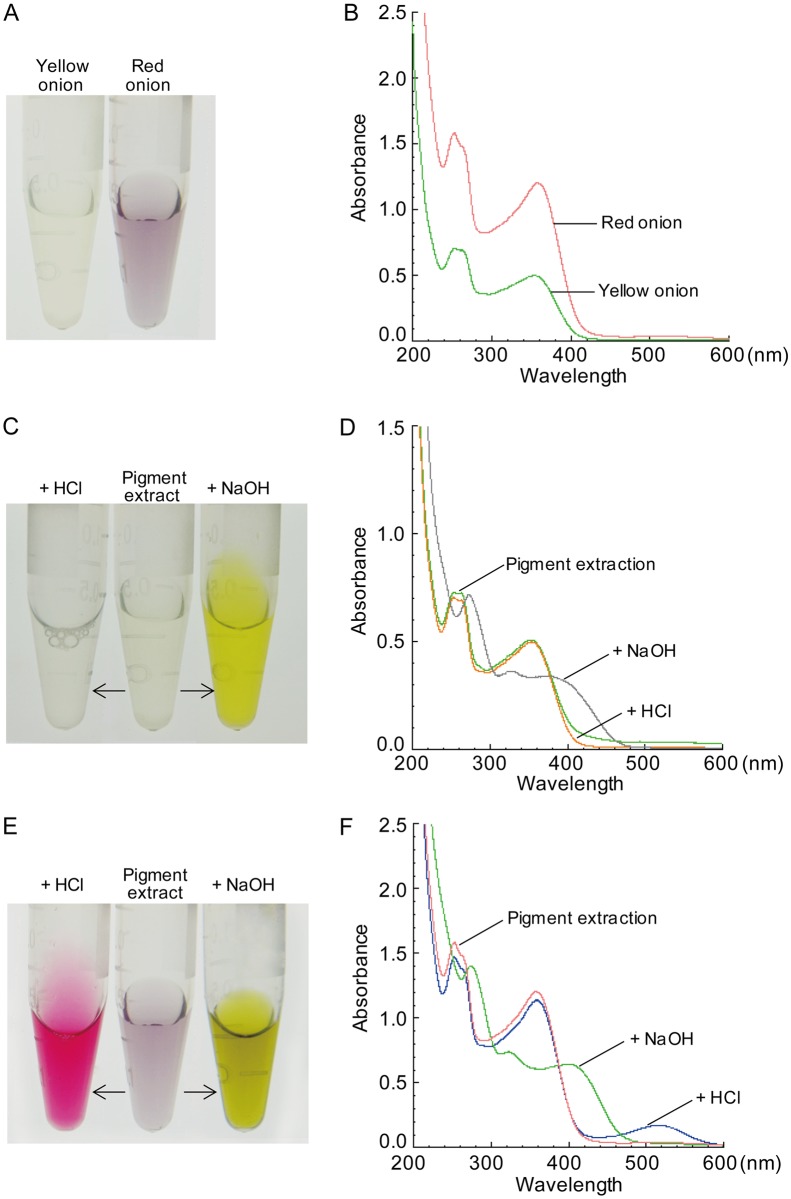
Comparison of pigment abundance and absorbance spectra of LE extracts between red and yellow onions. (A, B) The difference in color and spectra of pigment extracts of the LE between red and yellow onions. (C, D) Acid/base titration changed the color and spectra of LE pigment extracts from yellow onion scales. (E, F) Acid/base titration changed the color and spectra of LE pigment extracts from red onion scales.

Color differences existed between LE and UE of onion scales, as well as between red and yellow onion scales (Fig 3A). Onion pigments (mainly flavonoids) can be dertemined using high performance liquid chromatography-mass spectrometry, thin-layer chromatography, capillary electrophoresis, spectrophotometry and so on [19, 20]. Here we selected a relatively quick and easy spectrophotometry to detect the main compositions of onion pigments.

The absorbance spectra of onion LE extracts were characteristic of two major peaks at 255 nm and 363 nm for red onion, and 253 nm and 360 nm for yellow onion (Fig 3B). The two peaks possibly corresponded to quercetin, which has a typical absorbance peak at 255 nm and 370 nm [21]. A previous study determined that quercetin is the main flavonoid in two onion scales [7], and trace amounts of another flavonoid, anthocyanin, were also found in red onions [6].

It has been reported that pH affected the absorption peaks of quercetin and anthocyanin [21]. Thus, the spectra of LE pigment extracts were scanned after acid/base titration. At pH < 7, the light yellow of yellow onion extract became colorless; at pH >7, the color of the extract became dark yellow (Fig 3C). At pH < 7, the light purple of red onion extract became red; at pH >7, the color of the red onion extract became yellow (Fig 3E). At pH < 7, red onion extract had an additional absorption peak at 520 nm (Fig 3F), which was identified as anthocyanin. However, there was no 520 nm peak in yellow onion extract at pH < 7 (Fig 3D). Moreover, there were no obvious absorption peaks in pigment extracts of the UE from both red and yellow onions between 200–900 nm (results not shown). Our results showed that quercetin was abundant in the LE of red and yellow onion scales while anthocyanin was found in LE of red onion scales.

**Fig 4:**
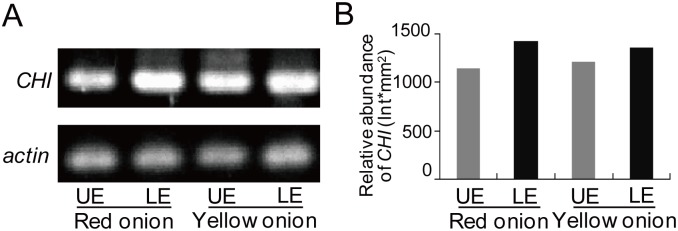
RT-PCR analysis of *CHI* expression in onion epidermises. (A) Gels of RT-PCR. (B) Relative abundance of *CHI* PCR products.

Comparative proteomic analysis revealed that two pigment synthesis-related proteins, chalcone-flavanone isomerase (CHI) (spots 28 and 43) and flavone O-methyltransferase 1-like (OMT1, spot 17), differentially accumulated between LE and UE (Table 3). CHI plays a part in the middle step of the flavonoid biosynthesis pathway by catalyzing the converse of chalcone into naringenin [22]. We checked the gene expression of *CHI* and found that it was up-regulated in LE compared with UE, especially in red onions (Fig 4), which was consistent with the abundance of this protein. The color difference between UE and LE and between red and yellow onions can be attributed to the differential accumulation of CHI, which increases flavonoid synthesis and the downstream production of quercetin and anthocyanin. OMT1, methylates 3’-OH residues in flavonoid compounds, such as quercetin, result in the formation of methoxy derivatives. Due to the effect of O-methylation on the solubility of flavonoids [23], highly abundant OMT1 in LE of red onions may contribute to the transformation of the tone of the onion by changing the solubility of flavonoids.

### Differential expression of stress-related proteins in onion epidermises

Both LE and UE of onion scales are the protective tissue, and play an important role in the process of various stress adaptation. In red onions, six stress-related proteins were more abundant in LE, whereas four were more abundant in UE (Table 3). Four stress-related proteins were the same between red and yellow onions, but showed abundance differences (Table 2).

The differentially accumulated proteins in LE of onions were previously proposed to resist various abiotic stresses (e.g., drought, cold, salt) [13, 24, 25] and biotic attacks (e.g., bacteria and fungi) [26, 27]. GDP-mannose 3',5'-epimerase 1 plays a protective role against reactive oxygen species, acid, drought and salt stresses in plants by regulating ascorbic acid biosynthesis [24]. In barley, formate dehydrogenase 1 responds to abiotic stresses (dark, chilling, drought, wounding) [28], and in pepper, it resists bacterial pathogens [26]. Momilactone A synthase-like and trans-resveratrol di-O-methyltransferase-like protect plants against pathogens by catalyzing the biosynthesis of plant phytoalexins, momilactone A [29] and pterostilbene [30]. Moreover, momilactone A synthase-like was induced to resist bacterial and fungal attacks in rice [31]. Chitinase, a member of pathogenesis related proteins (PRs), also enhanced disease resistance by degradation of chitin, which is the major component of fungal cell walls [27]. Peroxiredoxin-5 enhanced the resistance of abiotic and biotic stresses by catalyzing the decomposition of peroxides [32]. Tau glutathione S-transferase was found to participate in the freeze injury of onion scales [13], and the over-expressed *tau glutathione S-transferase* gene enhanced the resistance of transgenic tobacco to fluorodifen treatment, salt and drought [25]. Therefore, the above-mentioned seven highly abundant proteins in LE may contribute to the enhanced stress response of onion scales.

In the present study, such PRs as chitinase, β-1,3-glucanase and PR 5 were found to accumulate highly in UE of both onions but other PRs (e.g., intracellular PR and thaumatin-like protein 1b) were only in yellow onions (Table 3). Chitinase in red onions shown two species (spot 21 and 22) with a similar size but different p*I*s, which possibly results from post-translational modifications as found in tobacco [33]. Besides, PRs with diverse species were found to accumulate in UE of both onions. On one hand, PRs are known to protect plants against pathogens by attacking the molecules in the cell walls of bacteria or fungi or by spreading the ‘signal’ of the infection among plant cells [34]. PRs are known to promote the formation of local barricades in cell walls and slow the spread of pathogens in plants [35]. On the other hand, some PRs are also produced in response to abiotic stress. For instance, β-1,3-glucanase was up-regulated in response to aluminum toxicity [36], and thaumatin-like protein 1b enhanced plant tolerance to cold, drought, and salinity [37]. Salicylic acid-binding protein 2-like was reported to induce the production of PRs [38]. Hydroxyacylglutathione hydrolase cytoplasmic-like isoform X1 was up-regulated in response to abiotic stress in many plants [39]. For instance, an over-expression of the *hydroxyacylglutathione hydrolase cytoplasmic-like isoform X1* gene in tobacco enhanced salt tolerance [40].

### Epidermis differentiation and relevant differential proteins

Light microscopy showed that UE cells were larger (especially in width) than LE cells in both onions (Table 4). The ratio of length/width of LE cells in red onion was higher than that in yellow onion, and was also higher than that in UE cells of two onion types. Previously, the anisotropic expansion was found to be related to anisotropic cell wall structure and/or anisotropic stress distribution in the wall, suggesting cellulose microfibrils played an important role in anisotropic expansion [12]. Thus, the differences in cell size between UE and LE in both onion types may result from a combination of oriented cell divisions and anisotropic cell expansion.

**Table 4:** The average size of epidermis cells in red and yellow onion scales. Values were means ± SD (n = 3).

Cell Type		Length (μm)	Width (μm)
Red onion:	LE	29.28 ± 2.13 ^a^	6.20 ± 0.46^C^
	UE	31.69 ± 1.50 ^a^	13.19 ± 1.75^A^
Yellow onion:	LE	34.69 ± 3.77 ^a^	9.68 ± 1.90 ^B^
	UE	35.38 ± 3.07 ^a^	13.96 ± 1.32^A^

Note: Values having different superscripts (lowercase or majuscule) are significantly different. Lowercase indicates p<0.05 and majuscule indicates p<0.01.

**Fig 5:**
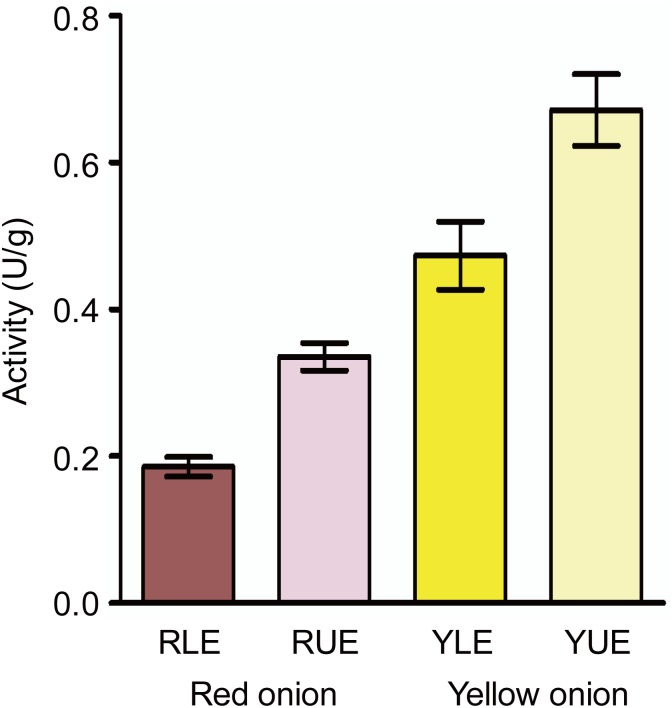
Comparison of β-1,3-glucanase activity in epidermises between red and yellow onions. The error bar indicates the standard deviation.

Comparative proteomic analysis revealed that two cell division related proteins, UDP-arabinopyranose mutase 1-like (UAM1, spot 18) and β-1,3-glucanase (spots 24, 25, 39 and 41), were differentially accumulated (Table 3). In the experiment of enzyme activity assay, the β-1,3-glucanase also showed a higher enzyme activity in UE than LE in both onions (Fig 5), which is consistent with the protein abundance. β-1,3-glucanase plays a vital role not only in defense mechanism of plants against pathogen infection, but in the growth and development of plants [41]. It catalyzes the decomposition of callose, which takes part in forming the cell-plate during cell division [42]. UAM1 participates in the essential reaction needed for the establishment of cell walls in plants [43]. The protein catalyzes the conversion of UDP-L-arabinopyranose to UDP-L-arabinofuranose. In *Arabidopsis*, non-expression of UAM1 causes several developmental retardation and dwarf phenotypes, and *UAM* knockout mutants produce approximately a 10% to 30% reduction of the monosaccharide content of the cell wall, which further restricts the formation of cell wall polysaccharide components [44]. Similarly, the differentially accumulated β-1,3-glucanase and UAM1 proteins in LE of red onion scales may result in smaller LE cells compared with LE cells of yellow onions and UE cells of both onions (Fig 1E–1H).

In addition, 13 differential proteins were involved in protein synthesis and carbohydrate metabolic processes, of which only 2,3-bisphosphoglycerat independent phosphoglycerate mutase and enolase existed in both onions with a similar abundance change (Table 3).

## Conclusions

In summary, comparative proteomic analysis in this study revealed that the proteome of UE and LE of onion scales were different. The differential proteins identified in the present study mainly participated in pigment synthesis (e.g., OMT1 and CHI), stress response (e.g., PRs), cell division (e.g., β-1,3-glucanase and UAM1), protein synthesis and carbohydrate metabolism. In addition, the differential proteins involved in cell division and stress response are worth to be further studied so as to reveal the mechanism of the differentiation and development of onion epidermis. The data derived from this study provides new insight into the differences in differentiation and developmental processes between onion epidermises, and may make a contribution to onion breeding, such as improving resistance and changing colors.

## Supporting Information

S1 Fig 2-DE detection of differentially abundant proteins between LE and UE in onion scales. CBB-stained gels from two independent experiments. Differential spots with at least two-fold changes in volume are indicated. A, B and C represent three independent replicates.
(PPT)

S1 File MS/MS information of 47 identified protein spots. (RAR)
