# Supplementary material for: Proteomic Characterization of Differential Abundant Proteins Accumulated between Lower and Upper Epidermises of Fleshy Scales in Onion (Allium cepa L.) Bulbs
Source: PLoS One. 2016 Dec 30;11(12):e0168959. doi: 10.1371/journal.pone.0168959 (PMC5201266; doi:10.1371/journal.pone.0168959)
Supplement: S1 Fig — CBB-stained gels from two independent experiments. Differential spots with at least two-fold changes in volume are indicated. A, B and C represent three independent replicates. (PPT) [file pone.0168959.s001.ppt]

## Slide 1
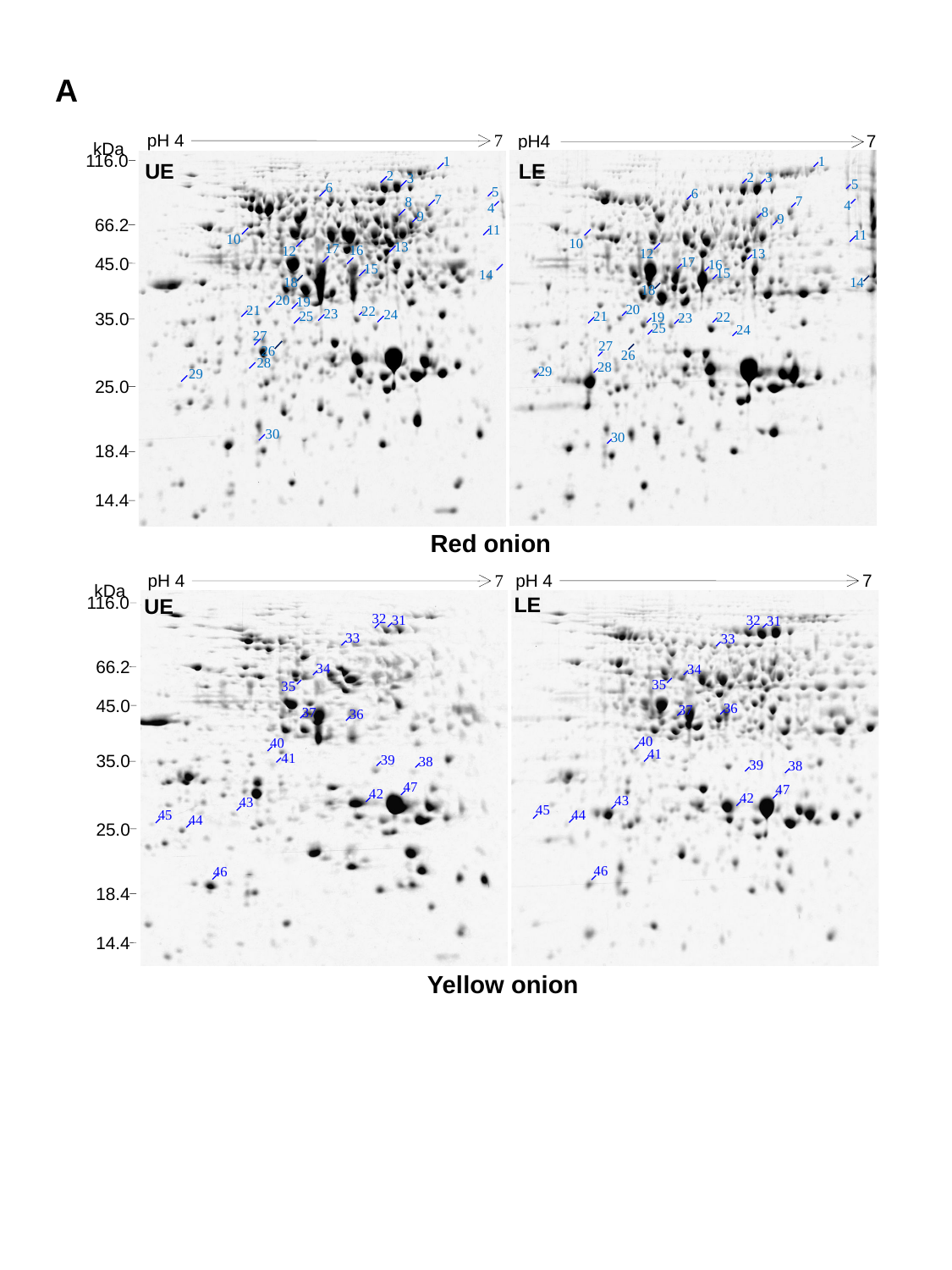

A
kDa
116.0
66.2
45.0
35.0
25.0
18.4
14.4
7
pH 4
pH4
7
1
LE
2
3
5
6
7
4
8
9
11
10
12
13
17
16
15
14
18
20
21
19
22
23
25
24
27
26
28
29
30
1
UE
2
3
6
5
7
8
4
9
11
10
13
17
16
12
15
14
18
20
19
21
22
23
24
25
27
26
28
29
30
Red onion
kDa
116.0
66.2
45.0
35.0
25.0
18.4
14.4
7
pH 4
pH 4
7
LE
UE
32
31
33
34
35
37
36
40
41
39
38
47
42
43
45
44
46
46
32
31
33
34
35
36
37
40
41
39
38
47
42
43
45
44
Yellow onion

## Slide 2
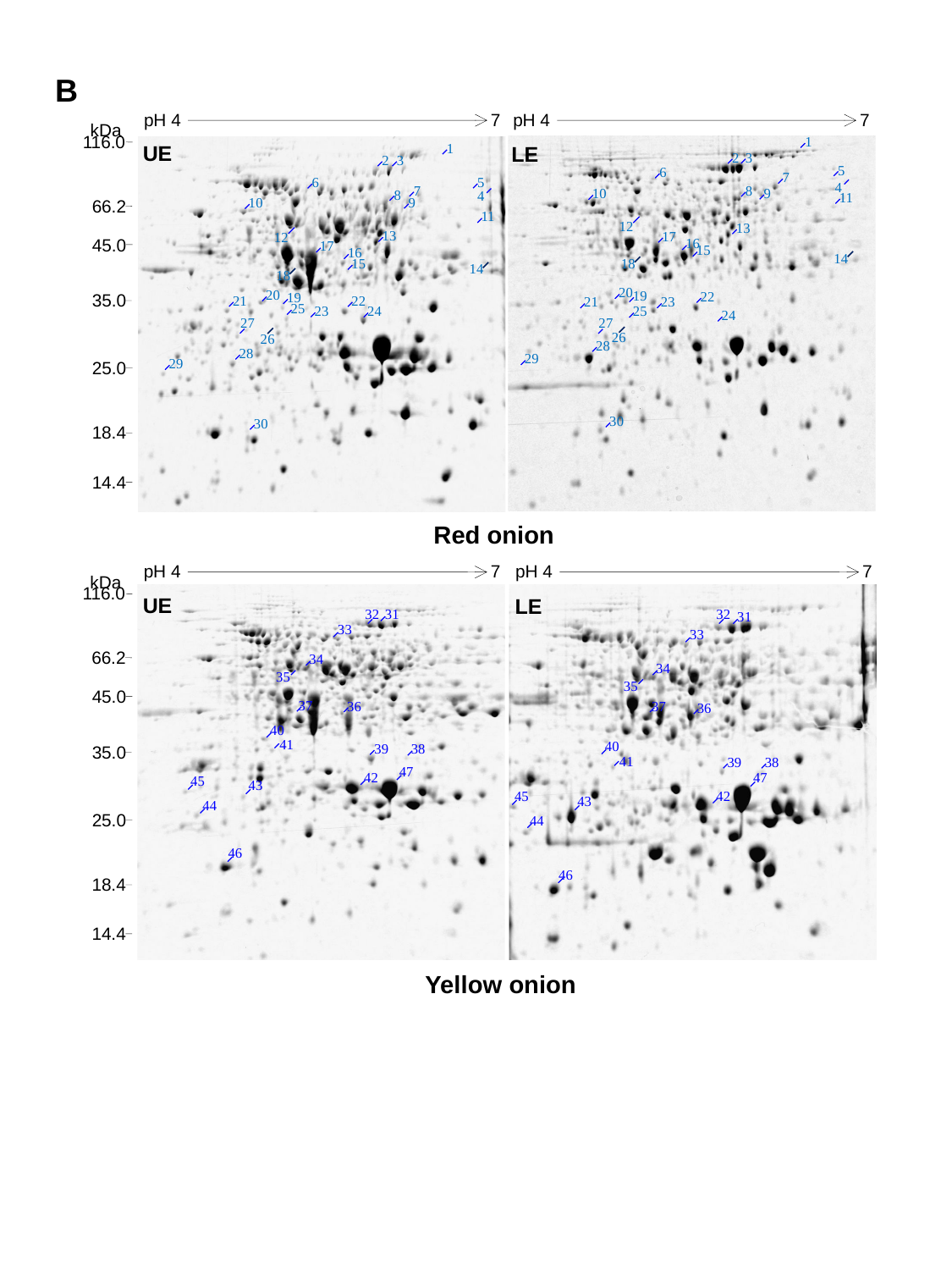

B
kDa
116.0
66.2
45.0
35.0
25.0
18.4
14.4
pH 4
7
pH 4
7
1
LE
2
3
5
6
7
4
8
10
9
11
12
13
17
16
15
14
18
20
19
22
21
23
25
24
27
26
28
29
30
1
UE
2
3
5
6
7
8
4
10
9
11
13
12
17
16
15
14
18
20
19
21
22
25
23
24
27
26
28
29
30
Red onion
kDa
116.0
66.2
45.0
35.0
25.0
18.4
14.4
pH 4
7
pH 4
7
UE
31
32
33
34
35
37
36
40
41
39
38
47
42
45
43
44
46
LE
32
31
33
34
35
37
36
40
41
39
38
47
42
45
43
44
46
Yellow onion

## Slide 3
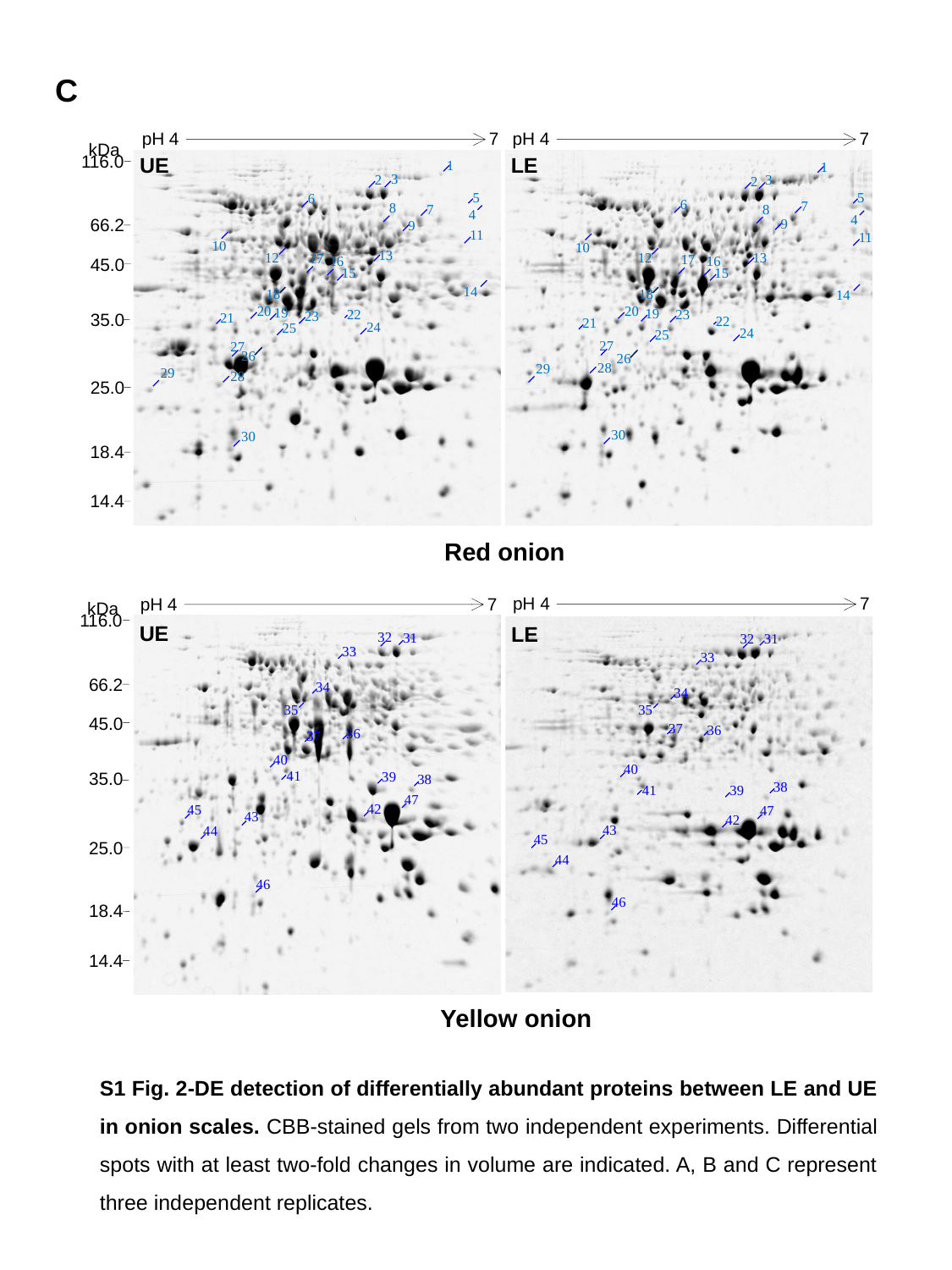

C
kDa
116.0
66.2
45.0
35.0
25.0
18.4
14.4
pH 4
7
pH 4
7
UE
1
3
2
5
6
8
7
4
9
11
10
13
12
17
16
15
14
18
20
19
22
23
21
24
25
27
26
29
28
30
LE
1
3
2
5
6
7
8
4
9
11
10
12
13
17
16
15
18
14
20
19
23
22
21
24
25
27
26
28
29
30
Red onion
kDa
116.0
66.2
45.0
35.0
25.0
18.4
14.4
pH 4
7
pH 4
7
UE
LE
32
31
33
34
35
36
37
40
41
39
38
47
42
45
43
44
46
32
31
33
34
35
37
36
40
38
41
39
47
42
43
45
44
46
Yellow onion
S1 Fig. 2-DE detection of differentially abundant proteins between LE and UE in onion scales. CBB-stained gels from two independent experiments. Differential spots with at least two-fold changes in volume are indicated. A, B and C represent three independent replicates.
